# PHYSICAL ACTIVITY MEDIATES THE EFFECTS OF DEPRESSIVE SYMPTOMS ON THE COGNITIVE FUNCTION OF OLDER ADULTS

**DOI:** 10.1093/geroni/igz038.1388

**Published:** 2019-11-08

**Authors:** Yit Mui Khoo, Hisako Matsuo

**Affiliations:** Saint Louis University, St. Louis, Missouri, United States

## Abstract

Evidence suggests that depressive symptoms among older adults were associated with cognitive impairment and affect cognitive decline over time, while physical activity was associated with lower risk of cognitive decline or have positive effect on cognitive function. The purpose of this study is to examine whether physical activity could mediate the effects of depressive symptoms on the cognitive function of older adults. Data from the 2014 Health and Retirement Survey (HRS) of older adults ≥ 60 years (N=9,753) were used. Hierarchical regression was conducted to examine the relationship between depressive symptoms, physical activity, and cognitive function. Mediation analysis was used to examine whether physical activity could mediate the effects of depressive symptoms on cognitive function. Regression results indicated that increased depressive symptoms was associated with poorer cognitive function, while increased moderate and mild physical activity were associated with better cognitive function. Mediation analysis indicated that the direct effect of depressive symptoms on cognitive function was significant. The indirect effect of depressive symptoms on cognitive function mediated by moderate and mild physical activity were also significant. Findings suggest that physical activity could potentially improve the cognitive function of older adults who have depressive symptoms. Moderate and mild physical activity could benefit older adults with depressive symptoms and reduce the risk of cognitive decline. Frail, disabled or chronically ill older adults are less likely to participate in vigorous physical activity, but they could benefit from moderate or mild physical activity and have better cognitive health.

